# The association between hallway boarding in internal wards, readmission and mortality rates: a comparative, retrospective analysis, following a policy change

**DOI:** 10.1186/s13584-021-00443-3

**Published:** 2021-01-27

**Authors:** Assaf Ben Shoham, Gabriel Munter

**Affiliations:** 1 Clalit Health Services, Jerusalem, Israel; 2 Shaare Zedek Medical Center, Jerusalem, Israel

**Keywords:** Boarding, Hallway beds, Internal department, Length of stay, 30-day readmission, 30-day mortality

## Abstract

**Background:**

Emergency department overcrowding is associated with adverse clinical outcomes and poor patients and staff experience. Full capacity protocols enabling hallway boarding in internal wards are instituted to relieve emergency room overcrowding. The effect of hallway boarding on the clinical outcomes of all inpatients in the internal wards has not been studied. Early in 2016, a decision to enable hallway boarding in the internal wing in our medical center came into effect, comprising an abrupt change to the medical center’s policy. The objective of this study is to examine the effect of hallway boarding on patients who were hospitalized in the internal wards.

**Methods:**

General linear regression analysis, based on administrative data about admissions of patients, from January 2013 through September 2019, is used to compare in-hospital mortality, 30-day readmission and 30-day mortality rates, of inpatients hospitalized in two internal departments in our medical center, before and after the policy change.

**Results:**

Eight thousand five hundred eighty-three patients and 11,962 patients were admitted to internal departments A and B, before and after the policy change, respectively. Adjusted in-hospital mortality was lower after the policy change (OR 0.76, [CI, 0.65 to 0.90]), 30-day readmission was mildly higher (OR, 1.18 [CI, 1.00 to 1.40]) and no change in 30-day mortality was observed (OR 1.16 [CI, 0.88 to 1.53]). The results emanate from corresponding changes in department A. No apparent change was observed in the length of hospital stay in department A, while a shorter length of stay was observed in department B.

**Conclusion:**

Enabling inpatient boarding in our medical center, effectively, had increased bed capacity and generated an increase in the volume of patients. It was associated with lower in-hospital mortality and an increased 30-day readmission, without increasing 30-day mortality. Since this is an observational study, conducted in a single center, further research is necessary to confirm and qualify these observations.

**Supplementary Information:**

The online version contains supplementary material available at 10.1186/s13584-021-00443-3.

## Background

Emergency department (ED) overcrowding is a concern for hospitals. It is associated with adverse clinical outcomes and poor patient and staff experience [1–3]. Insufficient inpatient bed capacity is an important contributor to ED overcrowding. Full capacity protocols (FCPs) specify conditions under which admitted patients may be transferred from the ED to temporary inpatient care spaces with the goal to safely share the burden of care of inpatients, improving clinical outcomes and mitigating the negative effects of ED overcrowding [1]. FCPs are widely practiced in North America as well as in many hospitals in Israel.

Previous research provides evidence about the effect of FCP on ED overcrowding [1]. Shorter ED boarding is associated with shorter overall hospital length of stay (LOS) and decreased hospital mortality, and transferring ED-boarded patients to inpatient hallway beds did not appear to result in patient harm [3, 4]. Patients themselves prefer hallway boarding in inpatient wards over ED boarding [5, 6]. There is lacking evidence however, regarding the effect of FCPs which enable hallway boarding on the clinical outcomes of all hospitalized patients in hallway-boarding wards, whether admitted to a hallway or to a room bed.

Shaare Zedek Medical Center (SZMC) is one of the largest hospitals in the area of Jerusalem, admitting over 7500 patients to the internal wards, annually. Until 2016 the medical center’s policy stipulated that admitted patients be transferred to inpatient internal wards only when an inpatient bed was available in one of the wards’ rooms. In particular, the practice of hallway boarding was shunned. Due to increasing number of admissions to SZMC’s ED and, subsequently, frequent and prolonged periods of ED overcrowding, a decision to employ a FCP that enables hallway boarding was implemented.

As in other hospitals, SZMC’s ED starts to fill up from mid-morning, whereas inpatient beds in the internal wards often become available late in the afternoon or early in the evening [1, 2]. SZMC adopted a protocol in which, every morning, ED staff, headed by a senior ED specialist, evaluates and assigns admitted patients to medical teams for further management and care. While some are held to board in the ED, select patients are transferred early in the morning to inpatient wards in the internal wing, where some are admitted to hallway beds. In principle, transferred patients were clinically stable, have had a formulated working diagnosis and a treatment plan prior to their transfer. In most days, hallway boarding patients were assigned to an inpatient bed in one of the wards’ rooms by the end of the same-day of admission. Each of the wards in the internal wing in the medical center’s main campus has 40 registered inpatient beds, and for the majority of time, throughout the year, is at full occupancy. Each of the wards was expected to board additional 4–7 admitted patients in the hallway. This protocol is similar to FCP described in the literature [4].

The objective of this research is to study the effect of the decision to enable inpatient hallway boarding in two departments in SZMC’s internal wing. We investigate the association between the timing when the decision took effect and clinical outcomes of all patients admitted to these departments, including in-hospital mortality, 30-day readmission and 30-day mortality rates following discharge.

## Methods

The data was extracted from the electronic medical records (EMR) of admissions to SZMC. We included admissions of patients who were both admitted and discharged from the internal wing during the study period, from January 2013 through September 2019, excluding patients who were transferred from the internal wing to other wings (e.g. general surgery), who account for less than 4% of all admissions to the internal wing (3.3 and 3.5%, before and after the policy change, respectively).

We included admissions to internal departments A and B, which had the same department head and experienced no changes for several years prior to and throughout the study period. We excluded admissions to internal departments C and D as well as Geriatrics, which, during the study period, had gone through changes, in either location, arrangement, number of inpatient beds, management practices or patient mix.

For each admission we extracted from the EMR, the date and time of admission, basic demographic data, active and background diagnoses and previous hospitalizations, and the date and time of in-hospital mortality or discharge. We identified subsequent admissions to SZMC within 30 days from discharge. However, we did not have data about subsequent admissions to other medical centers. For each patient admitted during the study period, we received from the ministry of health updated information about deaths, as of November 2019.

For each admission, we constructed the Charlson comorbidity index (CCI) [7, 8]. We calculated the LOS, i.e., the number of days from admission to discharge or death. 30-day readmission and mortality rates were calculated out of patients who were discharged (alive).

Comparisons of admissions’ characteristics, as well as outcome measures, were conducted with the appropriate tests (t-tests, Chi-squared, Pearson’s). To adjust outcomes for differences in admissions’ characteristics across the study periods, as well as for secular trends and seasonal variation, we conducted generalized linear multivariate (GLM) analysis, with the variable of interest being an indicator for the period after the decision to enable hallway boarding. The distribution of LOS, an important measure which is associated with hospitalization outcomes, is skewed to the right with a long tail. We thus used its logarithmic transformation in GLM analysis and incorporated its squared term to allow for non-linear association.

The analysis was performed using SPSS software, version 24 (SPSS, Chicago IL, USA). The study was approved by SZMC’s Review Board prior to data collection.

## Results

**Fig. 1:**
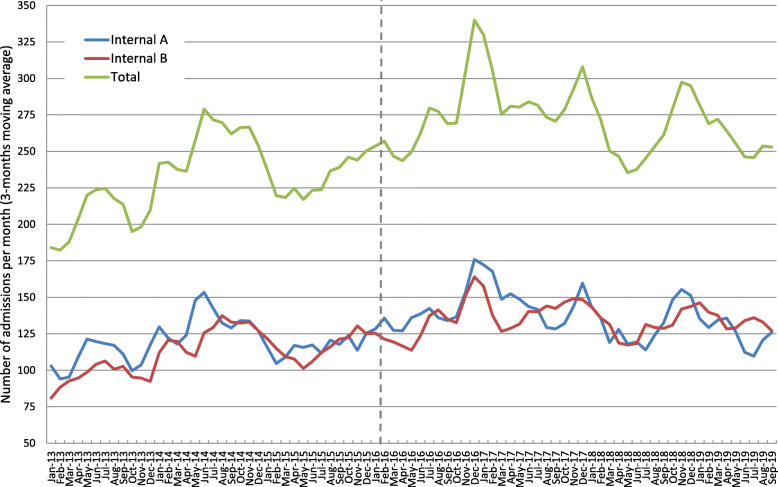
Number of admissions per month (3-months moving averages), by month, throughout the study period, by department

**Fig. 2:**
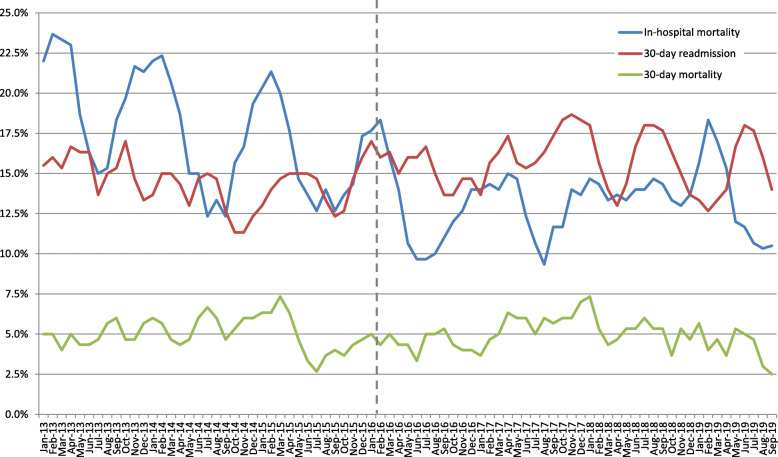
In-hospital mortality, 30-day readmission and 30-day mortality (3-months moving averages), by month, throughout the study period

Throughout the study period, 20,545 patients were admitted to internal departments A and B. The number of admissions per month, by department, is depicted in Fig. 1 (3-months moving averages). Similar trends are observed in both departments. The trends of the key outcome variables are depicted in Fig. 2 (3-months moving averages). Seasonality of in-hospital mortality rate is readily observed. A gradual decline of in-hospital mortality rate is also noted throughout 2013–2017 leveling off thereafter.

A total of 8583 patients were admitted prior to the policy change and a total of 11,962 patients were admitted afterwards. Admitted patients’ characteristics, before and after the policy change, were similar in each department (Table 1). Patients admitted to department A were slightly older and have had more hospitalizations during the year prior to the index admission The average number of admissions per month had increased by 14% in department A (from 120 to 137) and by 21% in department B (from 112 to 135). In turn, these changes resulted with an increase in the mean number of hospitalized patients, which had increased by 14% in department A (from 40 to 46 patients) and by 12% in department B (from 40 to 45 patients). The attenuated increase in the mean number of hospitalized patients in department B is compatible with the observed decrease in the LOS in this department, which was 0.7 days shorter after the policy change (*p*-value = 0.012).

**Table 1:** Characteristics of admissions before and after the policy change

Characteristics	Internal department A				Internal department B				All			
	Before	After	All	*p*-value	Before	After	All	*p*-value	Before	After	All	*p*-value
Number of admissions	4432	6028	10,460		4151	5934	10,085		8583	11,962	20,545	
Female (%)	53%	52%	53%	0.337	51%	52%	52%	0.115	52%	52%	52%	0.691
Age												
Mean (years)	77.9	77.5	77.7	0.165	75.5	76.3	76.0	0.011	76.7	76.9	76.8	0.417
Median (years)	82	81	81		80	80	80		81	81	81	
18–45 (%)	4%	4%	4%	0.247	6%	6%	6%	0.177	5%	5%	5%	0.629
45–64 (%)	12%	13%	12%		14%	13%	14%		13%	13%	13%	
65–74 (%)	15%	16%	15%		16%	17%	16%		16%	16%	16%	
75–84 (%)	30%	29%	29%		29%	28%	28%		29%	29%	29%	
85+ (%)	40%	39%	39%		35%	36%	36%		37%	38%	37%	
Hospitalization in the year prior to admission												
Mean (number)	2.0	2.0	2.0	0.898	1.9	1.9	1.9	0.117	1.9	2.0	2.0	0.334
0 (%)	33%	33%	33%	0.417	36%	34%	35%	0.044	34%	33%	34%	0.209
1–2 (%)	39%	39%	39%		37%	37%	37%		38%	38%	38%	
3–5 (%)	21%	20%	21%		19%	21%	20%		20%	21%	20%	
6+ (%)	8%	8%	8%		7%	8%	8%		8%	8%	8%	
Charlson Comorbidity Index												
CCI 0 (%)	28%	25%	26%	< 0.001	28%	28%	28%	0.080	28%	26%	27%	< 0.001
CCI 1–2 (%)	47%	46%	47%		45%	45%	45%		46%	46%	46%	
CCI 3–4 (%)	19%	22%	21%		21%	21%	21%		20%	21%	21%	
CCI > =5 (%)	5%	8%	7%		5%	7%	6%		5%	7%	6%	
Length of stay												
Mean (days)	10.2	10.2	10.2	0.962	10.9	10.2	10.5	0.012	10.5	10.2	10.3	0.072
SD (days)	13.4	14.0	13.7		15.3	13.4	14.2		14.4	13.7	14.0	
Median (days)	7	6	6		7	6	6		7	6	6	
0–1 (%)	5%	4%	4%	0.002	4%	4%	4%	0.001	5%	4%	4%	< 0.001
2–3 (%)	16%	18%	17%		16%	20%	18%		16%	19%	18%	
4–5 (%)	19%	20%	20%		20%	21%	21%		20%	21%	20%	
6–7 (%)	16%	17%	17%		16%	16%	16%		16%	16%	16%	
8–10 (%)	15%	15%	15%		14%	13%	14%		15%	14%	14%	
11–15 (%)	13%	11%	12%		12%	10%	11%		13%	11%	11%	
16–20 (%)	6%	5%	6%		6%	5%	6%		6%	5%	6%	
21–30 (%)	5%	5%	5%		6%	5%	5%		5%	5%	5%	
> 30 (%)	5%	5%	5%		6%	6%	6%		5%	5%	5%	
Number of admissions per month	120	137	129		112	135	125		232	272	254	
Number of hospitalized patients per day*	40	46	43		40	45	43		80	91	86	

* Calculated as the total number of hospitalization days divided by the number of days within the time-period

In-hospital mortality, 30-day readmission and 30-day mortality rates, before and after the policy change are exhibited in Table 2, unadjusted and adjusted by GLM regression analysis.

**Table 2:** In hospital mortality, 30-day readmission and 30-day mortality rates, by ward

	Unadjusted						GLM-adjusted*			
	Before	After	OR	CI - lower	CI-upper	*p*-value	OR	CI - lower	CI-upper	*p*-value
All†										
in-hospital mortality	17.5%	13.2%	0.718	0.665	0.775	< 0.001	0.764	0.649	0.898	0.001
30-day readmission	14.4%	15.7%	1.107	1.017	1.205	0.019	1.185	1.000	1.404	0.050
30-day mortality	5.0%	4.9%	0.975	0.849	1.12	0.722	1.159	0.876	1.534	0.302
Internal department A										
in-hospital mortality	18.1%	12.1%	0.63	0.56	0.70	< 0.001	0.736	0.584	0.927	0.009
30-day readmission	14.8%	16.3%	1.12	1.00	1.26	0.056	1.444	1.143	1.825	0.002
30-day mortality	5.2%	4.6%	0.87	0.72	1.06	0.174	0.904	0.606	1.349	0.622
Internal department B										
in-hospital mortality	16.9%	14.3%	0.82	0.74	0.92	< 0.001	0.798	0.634	1.003	0.053
30-day readmission	13.9%	15.0%	1.09	0.97	1.24	0.16	0.94	0.734	1.205	0.626
30-day mortality	4.9%	5.3%	1.09	0.89	1.33	0.398	1.459	0.983	2.166	0.061

*CI* Confidence interval, *OR* Odds ratio

*GLM logistic regression, adjusted by sex, age, Charlson Comorbidity Index, Number of hospitalization in the year prior to index admission, month dummies, time, log (LOS) and log (LOS) squared

† GLM logistic regression, adjusted also by ward

In-hospital mortality after the policy change was lower than before (OR 0.76). Analysis by department shows that the policy change was associated with a decrease of in-hospital mortality, in both departments, but was only significant in department A (OR 0.74, CI 0.58 to 0.93). The risk of in-hospital morality is strongly associated with older age, mildly associated with multiple co-morbidities and with multiple hospitalizations in the year prior to the index admission, and is increasingly greater with extended LOS (a convex association). In-hospital mortality exhibits seasonality, where higher mortality rates are associated with admissions during the winter months. A secular decrease in mortality was significant in department A (Table S1).

30-day readmission was higher than before (OR 1.18, CI 1.00 to 1.40). Analysis by department shows that the policy change was associated with a significant increase of 30-day readmission rate in department A (OR 1.44, CI 1.14 to 1.82). The risk of readmission at 30-days following discharge is associated with multiple hospitalizations in the year prior to the index admission, and mildly increasing with older age and with multiple co-morbidities. It is increasing with extended LOS. A secular decrease in rates of readmissions was found in department A (Table S2).

No significant change was observed in 30-day mortality, in both departments together, as well as separately following analysis by department. The risk of mortality within 30 days following discharge is strongly associated with older age, mildly associated with multiple co-morbidities and with multiple hospitalizations in the year prior to the index admission, and is increasingly greater with extended LOS (a convex association). 30-day mortality was not significantly higher in the winter months (Table S3).

## Discussion

The number of registered inpatient beds had not changed throughout the study period. Hallway boarding effectively increased the number of beds in each department, enabling higher capacity. The decision resulted with an increase in the number of admissions per month, a higher number of hospitalized patients per day and an increase in the burden of care. This observation concords with the fact that internal wards in Israel are a scarce resource, operating at full occupancy, throughout the year.

The policy change was associated with a significant decrease of the LOS in internal department B, estimated at 6%. Although the decrease in LOS may seem mild, at the department’s observed turn-over rate, it amounts, effectively, to an average of 2.75 inpatient beds less in the department. In internal department B, the policy change was thus associated with a higher turn-over rate and shorter LOS.

Shorter LOS may be associated with lower mortality rate via several mechanisms: the risk of hospital acquired infections is reduced; the risk of iatrogenic complications due to medical errors may be lower; de-conditioning and associated complications may be milder; alternatively, patients may have been discharged before medical stability was secured and thus in-hospital mortality rates may be lower while readmission and mortality rates after discharge may be higher. Our results show that although readmission rates increased after the policy change there was no significant difference in 30-day mortality rates.

The association between LOS and mortality is complex and is confounded by multiple factors. It is reasonable that complex medical conditions and patients with multiple co-morbidities are associated with both prolonged LOS and higher in-hospital mortality rates. Thus, at least is some range, LOS and in-hospital mortality may be positively correlated. Although it is also reasonable that very short LOS may be associated with increased 30-day mortality, such relationship is not plausible when considering in-hospital mortality, especially when considering similar medical conditions (clearly, some acute, severe conditions, may result in both very short LOS and high in-hospital mortality).

Some studies addressed the association between LOS, mortality and readmissions, among patients that were hospitalized due to specific medical condition. Miñana et al. studied the relationship of LOS with short-term readmission in patients who were hospitalized with acute heart failure (HF) [9]. They found that both short and long LOS were associated with increased risk of very early short-term readmission, defined as readmission within 10 days. Sud et al. examined the association between LOS, 30-day readmission and mortality rates among patients 65 years or older who were hospitalized with the diagnosis of HF [10]. They found a non-linear association between LOS and readmission, where short and long LOS were associated with increased rates of cardiovascular (CV) and HF associated readmissions (exhibiting U-shaped relationship). Long LOS was associated with increased rates of all-cause readmissions and mortality. Kaboli et al. follow LOS, 30-day readmission and mortality in US veteran hospitals over 14 years, during 1997–2010. During the study period mean LOS decreased from 5.44 to 3.98, where at the same time 30-day readmission rates fell from 16.5 to 13.8%, and all-cause 90-day mortality reduced by 3% annually [11].

The LOS in Israel is ranging low among OECD countries [12]. The LOS in internal medicine wards in the district of Jerusalem is the longest among Israel’s 6 districts (5.1 vs. 4.4 days nationally), and, among general hospitals in Jerusalem, is the longest in SZMC (6.7 days). In-hospital mortality rate in internal medicine wards in the district of Jerusalem was higher than average (5.2% vs. 5.0%), and, it was the second highest in the nation in SZMC (8.7%). Admittedly, these comparisons are not adjusted for patient mix. Indeed, SZMC was ranked first among 26 general hospitals in Israel, with respect to CCI of patients admitted to internal medicine wards. Admitted patients were, on average, older and had more diagnoses per patient in comparison to other hospitals in Israel [13]. It had the lowest percent of single day LOS (admission and discharge on the same day) and high 30-day readmission rates. The MOH conducted multivariate regression analysis to account for differences in patient mix and hospital characteristics. After adjustment, 30-day readmission rates in SZMC were found to be similar to the national average [13].

The above discussion may be relevant to the interpretation of the results in department B, where both a decrease in mortality and shorter LOS were observed. However, it is of little help in understanding the results in department A, where a decrease in mortality was observed with no apparent change in the LOS. With no actual changes in the department plan and infrastructure (e.g. toilets and showers, visitors’ area) and no changes in staffing, the effectively higher number of beds in each department may be associated with overcrowding and an increased risk of iatrogenic complications, which may be associated with an increased risk of in-hospital mortality. It is reasonable to hypothesize that an increased burden of care on medical staff may prolong the length of stay because less resources are allocated to each patient, and thus the observed LOS following the policy change is effectively shorter in comparison to the period prior to the change. This hypothesized qualitative decline in the effective LOS may be the driving force leading to the observed increase in 30-day readmission rate in department A.

As previous research provided evidence that shorter ED boarding is associated with shorter overall hospital LOS and decreased in-hospital mortality [3, 4], it may be that the outcomes observed in the internal wards in our study reflect these benefits. In this interpretation, the benefits of shorter ED boarding carry over to hospitalization in the internal wards, despite the increased burden of care in the wards and overcrowding, which may indeed hamper these benefits.

Compiling previous evidence in the literature, it may seem that implementing the FCP in SZMC was beneficial from the perspective of patients and medical staff in the ED, from the perspective of patients who were admitted to the internal wards, clinically as well as with respect to patients and ED staff experience and wellbeing. The policy change may also be financially desirable from the medical center’s perspective, since the remuneration scheme for purchasing services by the insurer is such that per-diem tariff is lower after the first 3 days of hospitalization, incentivizing higher turnover and shorter LOS while preserving full occupancy.

Lastly, it is important to emphasize again that no changes were made with respect to healthcare professional staffing within the internal wards thus the policy change is associated with increased workload and greater burden of care. It should be assumed, that pushing the medical staff to cope with an increased workload is associated with accelerated burnout, which may have detrimental effects on the wellbeing of medical staff and the quality of medical service they deliver [2, 14].

FCP enabling admission of patients to hallway beds has been criticized by nurses and medical professionals, as having a negative impact on patients and inpatient medical and nursing staff. Improvement in patient flow in and out of the ED may disrupt the safety and quality of care provision in the wards, where inpatient nurses and medical staff are expected to accept responsibility for the additional patients in hallway beds [14].

Recently, a committee for the improvement of treatment in internal departments in Israel, commissioned by the MOH, published its recommendations. The committee concurs that the practice of inpatient hallway boarding violates patients’ right for privacy and hampers human dignity. The committee recommends that such practice should be prohibited by a MOH circular, that additional internal wards be opened and existing wards renovated and adjusted to adequate standards of hospitalization [15].

This study has several limitations. First and foremost, a before-after analysis may be appropriate to evaluate the impact of policy change under strict conditions which were not plausibly satisfied in this study. In particular, one must convincingly assume that no other interventions or changes took place across the study time periods which may have had an effect on the outcome variables. SZMC is a dynamic hospital and changes that most certainly affect the standards of care continuously take place. Within the internal wing, some departments went through changes in the patients mix as well as adjustments in management practices. For this reason we excluded from the analysis departments that were clearly not adequate candidates for such before-after comparison; within the hospital, changes in the number and quality of medical staff, doctors and nurses, changes in safety and practices took place during the study period (e.g. the medical center went through an accreditation process during 2017 and preparations took place many months beforehand); across the hospital-ambulatory care interface, changes in programs to curb readmissions were implemented by Israel’s HMO’s during the study period. Nevertheless, it is difficult to disentangle the interaction between the trends over time and the partition of the study period into two time-periods within this study’s design. And, as mortality rates in both departments were gradually declining before the policy change, we cannot rule out the possibility that the policy change had an adverse effect on this trend. Second, readmission in our study is constructed on data restricted to hospitalization in SZMC alone. We did not have data on admissions to other hospitals following discharge from SZMC. Thus one should conclude that the reported 30-day readmission rates underestimate the true rates. Indeed, the MOH reports data about readmission to internal medicine wards. On average, across all general hospitals in Israel, 30-day readmission rates, to any hospital, were approximately 20% and 30-day readmission rates to the discharging hospital were approximately 17%, in each year during 2000–2015. Similar relative differences were found in SZMC [13, 16]. This however, may not undermine the qualitative comparison in our study since the same outcome measure is compared across the two time periods. Lastly, the results of this study, and in particular the association between hallway boarding and reduced in-hospital mortality, are not readily generalizable. We study select wards in a single center, during a particular time period, and further studies are necessary to substantiate and qualify general observations.

## Conclusions

This research is based on a large number of admissions to internal medicine departments, which, to our best judgment, employed similar practices throughout the study period. Our analysis addresses changes in the patients’ characteristics across the study periods. This study is unique in that it focuses on the association of hallway beds with outcome measure of inpatients in the internal wards taken as a whole, including patients who boarded in hallways and those who did not, allowing for spillover effects. It provides evidence and insight about the mechanism and the potential effects of employing a FCP as was adopted and implemented in SZMC.

Our results suggest that the policy introduced increased workload, and was associated with lower in-hospital mortality rate and an increased 30-day readmission rate without increasing the rate of 30-day mortality. This research provides weak evidence and more research is needed to study the impact of hallway beds on clinical outcome measures, as well as about the short and long term impact of this policy on medical stuff burnout and wellbeing.

## Supplementary Information

**Additional file 1.**

## Abbreviations

GLM: Generalized linear regression
LOS: Length of stay
SD: Standard deviation
OR: Odds ratio
ED: Emergency department
FCP: Full capacity protocol
SZMC: Shaare Zedek medical center
CCI: Charlson comorbidity index
HF: Heart failure
CV: Cardio vascular
