# Supplementary material for: The association between hallway boarding in internal wards, readmission and mortality rates: a comparative, retrospective analysis, following a policy change
Source: Isr J Health Policy Res. 2021 Jan 27;10:8. doi: 10.1186/s13584-021-00443-3 (PMC7842011; doi:10.1186/s13584-021-00443-3)
Supplement: Supplementary file 1 — Additional file 1. [file 13584_2021_443_MOESM1_ESM.docx]

Table S1: In-hospital mortality, GLM regression analysis

|  | Internal department A | |  | Internal department B | |  | All | |
| --- | --- | --- | --- | --- | --- | --- | --- | --- |
| Characteristics | Exp(B)* | p-value |  | Exp(B)* | p-value |  | Exp(B)* | p-value |
| Intercept | 1.006 | 0.983 |  | 0.247 | <0.001 |  | 0.475 | <0.001 |
| Female | 0.918 | 0.146 |  | 0.958 | 0.463 |  | 0.938 | 0.124 |
| Age |  |  |  |  |  |  |  |  |
| 45 – 64 | 1.367 | 0.200 |  | 2.593 | <0.001 |  | 1.950 | <0.001 |
| 65 – 74 | 1.705 | 0.025 |  | 3.566 | <0.001 |  | 2.566 | <0.001 |
| 75 – 84 | 2.495 | <0.001 |  | 4.515 | <0.001 |  | 3.470 | <0.001 |
| 85+ | 4.475 | <0.001 |  | 6.636 | <0.001 |  | 5.640 | <0.001 |
| Hospitalization in the year prior to admission | | | |  |  |  |  |  |
| 1 – 2 | 0.974 | 0.706 |  | 1.390 | 0.000 |  | 1.162 | 0.002 |
| 3 – 5 | 1.099 | 0.247 |  | 1.254 | 0.007 |  | 1.175 | 0.005 |
| 6+ | 1.035 | 0.770 |  | 1.416 | 0.002 |  | 1.216 | 0.016 |
| Charlson Comorbidity Index | |  |  |  |  |  |  |  |
| CCI 1-2 | 1.168 | 0.042 |  | 1.215 | 0.012 |  | 1.192 | 0.001 |
| CCI 3-4 | 1.468 | <0.001 |  | 1.567 | <0.001 |  | 1.511 | <0.001 |
| CCI >=5 | 2.011 | <0.001 |  | 2.224 | <0.001 |  | 2.097 | <0.001 |
| LOG LOS | 0.098 | <0.001 |  | 0.112 | <0.001 |  | 0.105 | <0.001 |
| LOG LOS Squared | 1.676 | <0.001 |  | 1.631 | <0.001 |  | 1.651 | <0.001 |
| Internal department B |  |  |  |  |  |  | 1.090 | 0.035 |
| Month of admission |  |  |  |  |  |  |  |  |
| February | 0.891 | 0.362 |  | 1.101 | 0.477 |  | 0.972 | 0.755 |
| March | 0.926 | 0.542 |  | 1.266 | 0.074 |  | 1.054 | 0.564 |
| April | 0.634 | <0.001 |  | 1.001 | 0.992 |  | 0.780 | 0.009 |
| May | 0.585 | <0.001 |  | 1.174 | 0.241 |  | 0.814 | 0.027 |
| June | 0.513 | <0.001 |  | 0.687 | 0.010 |  | 0.586 | <0.001 |
| July | 0.596 | <0.001 |  | 0.723 | 0.021 |  | 0.644 | <0.001 |
| August | 0.546 | <0.001 |  | 0.720 | 0.022 |  | 0.614 | <0.001 |
| September | 0.613 | <0.001 |  | 0.724 | 0.026 |  | 0.655 | <0.001 |
| October | 0.609 | <0.001 |  | 1.010 | 0.946 |  | 0.767 | 0.007 |
| November | 0.660 | 0.002 |  | 0.932 | 0.619 |  | 0.770 | 0.007 |
| December | 0.694 | 0.005 |  | 1.083 | 0.558 |  | 0.850 | 0.083 |
| Time | 0.995 | 0.040 |  | 1.000 | 0.970 |  | 0.997 | 0.153 |
| After | 0.736 | 0.009 |  | 0.798 | 0.053 |  | 0.764 | 0.001 |
|  |  |  |  |  |  |  |  |  |
| Omnibus Test (likelihood ratio) | 785.8 | |  | 715.9 | |  | 1439.9 | |
| p-value | <0.001 | |  | <0.001 | |  | <0.001 | |

*Coefficients (Exp(B)) in a logistic regression represent odds-ratios

Table S2: 30-day readmission, GLM regression analysis

|  | Internal department A | |  | Internal department B | |  | All | |
| --- | --- | --- | --- | --- | --- | --- | --- | --- |
| Characteristics | Exp(B)* | p-value |  | Exp(B)* | p-value |  | Exp(B)* | p-value |
| Intercept | 0.012 | <0.001 |  | 0.010 | <0.001 |  | 0.011 | <0.001 |
| Female | 0.978 | 0.708 |  | 0.874 | 0.036 |  | 0.927 | 0.086 |
| Age |  |  |  |  |  |  |  |  |
| 45 – 64 | 1.316 | 0.174 |  | 1.066 | 0.732 |  | 1.184 | 0.216 |
| 65 – 74 | 1.426 | 0.074 |  | 1.064 | 0.738 |  | 1.234 | 0.118 |
| 75 – 84 | 1.551 | 0.022 |  | 1.501 | 0.019 |  | 1.509 | 0.001 |
| 85+ | 1.880 | 0.001 |  | 1.857 | <0.001 |  | 1.849 | <0.001 |
| Hospitalization in the year prior to admission | | | |  |  |  |  |  |
| 1 – 2 | 1.668 | <0.001 |  | 1.546 | <0.001 |  | 1.609 | <0.001 |
| 3 – 5 | 2.542 | <0.001 |  | 2.480 | <0.001 |  | 2.506 | <0.001 |
| 6+ | 3.851 | <0.001 |  | 3.541 | <0.001 |  | 3.703 | <0.001 |
| Charlson Comorbidity Index | |  |  |  |  |  |  |  |
| CCI 1-2 | 1.235 | 0.009 |  | 1.208 | 0.024 |  | 1.224 | <0.001 |
| CCI 3-4 | 1.600 | <0.001 |  | 1.481 | <0.001 |  | 1.550 | <0.001 |
| CCI >=5 | 1.446 | 0.004 |  | 1.302 | 0.064 |  | 1.385 | 0.001 |
| LOG LOS | 2.983 | <0.001 |  | 3.383 | <0.001 |  | 3.116 | <0.001 |
| LOG LOS Squared | 0.849 | <0.001 |  | 0.848 | <0.001 |  | 0.852 | <0.001 |
| Internal department B |  |  |  |  |  |  | 0.957 | 0.309 |
| Month of admission |  |  |  |  |  |  |  |  |
| February | 1.103 | 0.507 |  | 0.866 | 0.356 |  | 0.982 | 0.865 |
| March | 1.048 | 0.752 |  | 0.934 | 0.661 |  | 0.989 | 0.918 |
| April | 0.920 | 0.569 |  | 1.042 | 0.787 |  | 0.977 | 0.826 |
| May | 1.075 | 0.607 |  | 0.929 | 0.644 |  | 1.006 | 0.955 |
| June | 1.001 | 0.997 |  | 1.057 | 0.710 |  | 1.031 | 0.769 |
| July | 1.285 | 0.075 |  | 1.100 | 0.512 |  | 1.194 | 0.080 |
| August | 1.215 | 0.166 |  | 0.842 | 0.264 |  | 1.027 | 0.800 |
| September | 0.968 | 0.826 |  | 0.996 | 0.977 |  | 0.981 | 0.856 |
| October | 1.145 | 0.364 |  | 0.968 | 0.836 |  | 1.058 | 0.605 |
| November | 1.026 | 0.865 |  | 1.040 | 0.802 |  | 1.025 | 0.818 |
| December | 0.977 | 0.878 |  | 0.982 | 0.904 |  | 0.987 | 0.903 |
| Time | 0.994 | 0.013 |  | 1.004 | 0.177 |  | 0.998 | 0.346 |
| After | 1.444 | 0.002 |  | 0.940 | 0.626 |  | 1.185 | 0.050 |
|  |  |  |  |  |  |  |  |  |
| Omnibus Test (likelihood ratio) | 395.8 | |  | 422.2 | |  | 792.9 | |
| p-value | <0.001 | |  | <0.001 | |  | <0.001 | |

*Coefficients (Exp(B)) in a logistic regression represent odds-ratios

Table S3: 30-day mortality, GLM regression analysis

|  | Internal department A | |  | Internal department B | |  | All | |
| --- | --- | --- | --- | --- | --- | --- | --- | --- |
| Characteristics | Exp(B)* | p-value |  | Exp(B)* | p-value |  | Exp(B)* | p-value |
| Intercept | 0.001 | <0.001 |  | 0.001 | <0.001 |  | 0.001 | <0.001 |
| Female | 1.088 | 0.416 |  | 0.873 | 0.183 |  | 0.975 | 0.729 |
| Age |  |  |  |  |  |  |  |  |
| 45 – 64 | 2.581 | 0.121 |  | 1.617 | 0.289 |  | 1.935 | 0.068 |
| 65 – 74 | 2.445 | 0.141 |  | 1.638 | 0.271 |  | 1.900 | 0.073 |
| 75 – 84 | 4.532 | 0.011 |  | 3.665 | 0.002 |  | 3.884 | <0.001 |
| 85+ | 6.961 | 0.001 |  | 5.387 | <0.001 |  | 5.816 | <0.001 |
| Hospitalization in the year prior to admission | | | |  |  |  |  |  |
| 1 – 2 | 1.151 | 0.272 |  | 1.207 | 0.144 |  | 1.177 | 0.072 |
| 3 – 5 | 1.188 | 0.244 |  | 1.532 | 0.002 |  | 1.359 | 0.002 |
| 6+ | 2.042 | <0.001 |  | 1.620 | 0.011 |  | 1.835 | <0.001 |
| Charlson Comorbidity Index | |  |  |  |  |  |  |  |
| CCI 1-2 | 1.827 | <0.001 |  | 1.270 | 0.106 |  | 1.512 | <0.001 |
| CCI 3-4 | 2.703 | <0.001 |  | 2.169 | <0.001 |  | 2.409 | <0.001 |
| CCI >=5 | 3.457 | <0.001 |  | 3.182 | <0.001 |  | 3.294 | <0.001 |
| LOG LOS | 2.956 | 0.002 |  | 5.114 | <0.001 |  | 3.903 | <0.001 |
| LOG LOS Squared | 0.889 | 0.078 |  | 0.819 | 0.002 |  | 0.853 | 0.001 |
| Internal department B |  |  |  |  |  |  | 1.162 | 0.034 |
| Month of admission |  |  |  |  |  |  |  |  |
| February | 1.372 | 0.185 |  | 0.639 | 0.062 |  | 0.930 | 0.666 |
| March | 1.015 | 0.952 |  | 0.488 | 0.007 |  | 0.702 | 0.051 |
| April | 0.888 | 0.640 |  | 1.005 | 0.983 |  | 0.953 | 0.774 |
| May | 1.211 | 0.416 |  | 0.822 | 0.418 |  | 0.984 | 0.924 |
| June | 0.797 | 0.390 |  | 0.807 | 0.345 |  | 0.811 | 0.220 |
| July | 1.245 | 0.362 |  | 0.676 | 0.094 |  | 0.906 | 0.551 |
| August | 1.271 | 0.309 |  | 0.994 | 0.977 |  | 1.115 | 0.495 |
| September | 1.020 | 0.937 |  | 0.753 | 0.227 |  | 0.867 | 0.403 |
| October | 1.028 | 0.915 |  | 0.738 | 0.225 |  | 0.855 | 0.382 |
| November | 1.044 | 0.865 |  | 0.842 | 0.471 |  | 0.920 | 0.628 |
| December | 1.267 | 0.321 |  | 0.903 | 0.649 |  | 1.065 | 0.697 |
| Time | 0.998 | 0.654 |  | 0.993 | 0.101 |  | 0.995 | 0.125 |
| After | 0.904 | 0.622 |  | 1.459 | 0.061 |  | 1.159 | 0.302 |
|  |  |  |  |  |  |  |  |  |
| Omnibus Test (likelihood ratio) | 232.8 | |  | 307.8 | |  | 510.5 | |
| p-value | <0.001 | |  | <0.001 | |  | <0.001 | |

*Coefficients (Exp(B)) in a logistic regression represent odds-ratios
